# Hainanenin-1, an oncolytic peptide, triggers immunogenic cell death via STING activation in triple-negative breast cancer

**DOI:** 10.1186/s12964-024-01731-6

**Published:** 2024-07-05

**Authors:** Xiaoxi Li, Nan Su, Haining Yu, Xiaoyan Li, Shu-lan Sun

**Affiliations:** 1 Central Laboratory, Cancer Hospital of Dalian University of Technology, Cancer Hospital of China Medical University (Liaoning Cancer Hospital & Institute), Shenyang, Liaoning 110042 P. R. China; 2 School of Bioengineering, Dalian University of Technology, Dalian, Liaoning 116024 P. R. China; 3 Department of Pathology, Cancer Hospital of Dalian University of Technology, Cancer Hospital of China Medical University (Liaoning Cancer Hospital & Institute), Shenyang, Liaoning 110042 P. R. China; 4 Central Laboratory, Cancer Hospital of Dalian University of Technology (Liaoning Cancer Hospital & Institute), Shenyang, Liaoning 110042 P. R. China

**Keywords:** Host defense peptide, Immunogenic cell death, STING, Triple-negative breast cancer, Anti-tumor immunity, HN-1

## Abstract

**Background:**

In triple-negative breast cancer (TNBC) therapy, insufficient tumor infiltration by lymphocytes significantly hinders the efficacy of immune checkpoint inhibitors. We have previously demonstrated that Hainanenin-1 (HN-1), a host defense peptide (HDP) identified from Hainan frog skin, induces breast cancer apoptosis and boots anti-tumor immunity via unknown mechanism.

**Methods:**

We used in vitro experiments to observe immunogenic cell death (ICD) indicators in HN-1-treated TNBC cell lines, a mouse tumor model to verify HN-1 promotion of mice anti-tumor immune response, and an in vitro drug sensitivity test of patient-derived breast cancer cells to verify the inhibitory effect of HN-1.

**Results:**

HN-1 induced ICD in TNBC in a process during which damage-associated molecular patterns (DAMPs) were released that could further increase the anti-tumor immune response. The secretion level of interleukin 2 (IL-2), IL-12, and interferon γ in the co-culture supernatant was increased, and dendritic cells (DCs) were activated via a co-culture with HN-1-pretreated TNBC cells. As a result, HN-1 increased the infiltration of anti-tumor immune cells (DCs and T lymphocytes) in the mouse model bearing both 4T1 and EMT6 tumors. Meanwhile, regulatory T cells and myeloid-derived suppressor cells were suppressed. In addition, HN-1 induced DNA damage, and double-strand DNA release in the cytosol was significantly enhanced, indicating that HN-1 might stimulate ICD via activation of STING pathway. The knockdown of STING inhibited HN-1-induced ICD. Of note, HN-1 exhibited inhibitory effects on patient-derived breast cancer cells under three-dimensional culture conditions.

**Conclusions:**

Collectively, our study demonstrated that HN-1 could be utilized as a potential compound that might augment immunotherapy effects in patients with TNBC.

**Supplementary Information:**

The online version contains supplementary material available at 10.1186/s12964-024-01731-6.

## Background

Globally, breast cancer is the second most prevalent cause of cancer-related death among women [1], and the proportion of triple-negative breast cancer (TNBC) cases ranges between nearly 10% and 20% [2]. High invasion and metastasis in the early stage of cancer are the key characteristics of TNBC, with a poor prognosis and more than 50% of patients relapsing within 3 to 5 years after diagnosis and treatment [3]. Available endocrine or HER2-targeted drugs for patients with TNBC have limited efficacy because they lack estrogen receptor (ER), progesterone receptor (PR), and human epidermal growth factor receptor 2 (HER2) expression. The systemic treatment of TNBC involves a chemical treatment, but the treatment effect is not ideal. The median overall survival (OS) period is 13 to 18 months [4]. In contrast to other molecular types of breast cancer, TNBC shows higher PD-L1 expression and immune cell infiltration, suggesting that patients with TNBC may benefit from immunotherapy, despite its limited efficacy due to the lack of infiltrating effector lymphocytes [5]. Therefore, an increasing number of studies tend to combine immunotherapy with other therapies that could stimulate the anti-tumor immune responses.

In adaptive immunity, T lymphocytes are very important effector cells; therefore, comprehending their activation mechanism has become essential in cancer immunotherapy [6]. Several studies have shown that several anticancer drugs release damage-associated molecular patterns (DAMPs) and tumor-related antigens, thus inducing tumor cell immunogenic cell death (ICD), dendritic cell (DC) maturation, and cytotoxic T lymphocyte (CTL) infiltration [7, 8]. DAMPs, as markers of ICD, include ATP, calreticulin (CRT), heat shock proteins (HSPs), and high-mobility group box 1 (HMGB1) [9]. The expression of CRT on the surface of tumor cells can help DCs recognize dead cells by presenting them as “eat me” signals [10]. The release of HMGB1 leads to the production of proinflammatory factors, including interleukin (IL) 1, IL-12, tumor necrosis factor (TNF), and type I interferon (IFN). In addition to producing type I IFN, dead cells secrete CXCL10, which is involved in the chemotaxis of T cells toward the tumor microenvironment [11]. These danger signals can initiate adaptive immune responses targeting tumor cells but also improve immunotherapy efficacy by reversing the immunosuppressive tumor microenvironment [12].

A class of conserved innate immune factors are host defense peptides (HDPs), which play key roles in promoting immunity and regulating the immune response [13]. HDPs, including cecropin B, melittin, BMAP-28, magainin, lactoferrin, and human catalytin-LL37, have strong anti-tumor properties both in vivo and in vitro [14, 15]. There is also evidence suggesting that some natural HDPs induce the activation and differentiation of macrophages, T lymphocytes, and DCs and effectively inhibit tumor cell proliferation [16, 17]. Hainanenin-1 (HN-1) is a naturally produced HDP from the skin of the Hainan frog (Amolops hainanensis) [18, 19]. In our previous work, we found that HN-1 inhibited tumor growth and induced immune cell infiltration; however, its underlying mechanism is still unknown [20].

In the study hereby described, we discovered that HN-1 induced TNBC cell line ICD and triggered DC maturation. In a mouse tumor model, HN-1 significantly inhibited 4T1 tumor growth and increased the infiltration of anti-tumor immune cells (DCs and CD4^+^ and CD8^+^ T lymphocytes), while the proportion of suppressive immune cells (regulatory T (Treg) cells and polymorphonuclear myeloid-derived suppressor cells (PMN-MDSCs)) was significantly reduced. Furthermore, we found that HN-1 treatment induced double-strand DNA (dsDNA) release and triggered ICD via activation of-STING pathway. Knocking down STING with si-STING inhibited the HN-1-induced ICD response. Of note, HN-1 exhibited inhibitory effects on patient-derived breast cancer cells under three-dimensional culture conditions. Monotherapy and the simple induction of cell apoptosis to kill cancer cells are not especially effective in the treatment of cancer. Cancer treatment methods such as chemotherapy and radiation combined with immunotherapy can induce anti-tumor immunity effectively, thereby eradicating tumors and generating long-term anti-tumor immune responses [21]. Therefore, HN-1 could be a treatment option for patients with TNBC that might augment immunotherapy efficacy.

## Methods

### Reagent

HN-1, a 21 mer peptide (FALGAVTKLLPSLLCMITRKC), was synthesized and purified by Sangon Biotech Co., Ltd. (Shanghai, China) using reverse-phase high-performance liquid chromatography and electronic spray ionization mass spectrometry to attain ~ 95% purity (Supplementary Fig. 1). BI-6C9 was obtained from MedChemexpress (MCE, New Jersey, USA). Both the anti-CD8α antibody and the isotype IgG1 antibody were purchased from BioXcell (New Hampshire, USA).

### Cell culture

The human breast cancer cell lines MDA-MB-453 and MDA-MB-231 and the mouse breast cancer cell lines 4T1 and EMT6 were purchased from the Cell Bank of the Chinese Academy of Sciences (Shanghai, China). MDA-MB-453 and MDA-MB-231 were maintained in L-15 medium (HyClone, GE Healthcare Life Sciences, Logan, UT, USA) supplemented with 10% fetal bovine serum (FBS, Gibco, Thermo Fisher Scientific, Inc., Waltham, MA, USA). The 4T1 and EMT6 cells were cultured in RPMI-1640 supplemented with 10% fetal bovine serum.

### Immunofluorescence staining

The MDA-MB-231 and MDA-MB-453 cells were treated with 20 µM HN-1, then fixed with 95% ethanol, and permeated with PBS containing 1% Triton X-100. Next, the cells were incubated with an anti-calreticulin monoclonal antibody (1:75; ab2907; Abcam), a dsDNA monoclonal antibody (1:50; 15,635; Cayman Chemical), or Photoshop Histone H2A.X (1:200; 9718; Cell Signaling Technology) overnight at 4 °C. The cells were washed three times with PBS and then incubated with the secondary antibody goat anti-rabbit Alexa Fluor 488 (1:200; ab150077; Abcam) or goat anti-mouse Alexa Fluor 488 (1:200; ab150113; Abcam) for an additional 30 min, after which Hoechst 33,342 was used to stain the cell nucleus. Finally, the cells were observed by confocal fluorescence microscopy (Olympus, FV1000, Tokyo, Japan), and the ImageJ software (version 1.51j8; National Institutes of Health and University of Wisconsin, Bethesda, MD, USA) was used for quantification.

### DC culture

We recruited two female volunteers, aged 30 and 38 years, who provided informed consent. Isolated peripheral blood mononuclear cells (PBMCs) were stained with FITC-conjugated HLA-A2 (343,304; Biolegend, San Diego, CA, USA), and human leukocyte antigen (HLA) subtypes were detected via flow cytometry. The PBMCs were cultured in 75 cm2 cell culture flasks for 1 h. After removing the suspended cells, the adherent cells were cultured in fresh X-VIVO 15 medium (Lonza, Alpharetta, GA, USA) supplemented with 5% plasma, 500 U/ml IL-4, and 1000 U/ml GM-CSF (Promega, Madison, WI, USA). After 5 days of stimulation, the DCs were collected for further investigation.

### Enzyme-linked immunosorbent assays (ELISAs)

The MDA-MB-231 and MDA-MB-453 cells were treated with 20 µM HN-1, and the culture supernatant was collected to detect the secretion of ATP (Promega, Madison, WI, USA) and HMGB1 (Signalway Antibody, MD, USA). The MDA-MB-231 cells were pretreated with HN-1 and co-cultured with DCs for 48 h, after which the IL-2, IL-10, IL-12, and IFN-γ levels were detected. The quantification of cytokines in the supernatant was performed using a human ELISA detection kit (IL-2, D2050; IL-10, D1000B, IFN-γ, DIF50C; R&D Systems, MN, USA) in accordance with the instructions provided by the manufacturer.

### Mouse tumor model

All the procedures were performed according to protocols approved by China Medical University’s Institutional Committee for the Use and Care of Laboratory Animals. Five-week-old female BALB/c mice or BALB/c nude mice were obtained from Vital River Laboratories (Beijing, China). For the immunocompetent mice study, 2 × 10^5^ 4T1 or EMT6 mouse breast cancer cells were inoculated into mammary fat pads. For the nude mice study, 3 × 10^6^ MDA-MB-231 or MDA-MB-453 cells were implanted into mammary fat pads. After the long and short diameters of the tumors on both sides reached 5 mm, the mice were randomly divided into a control group and an experimental group. The control group of mice was intraperitoneally injected with PBS, while the HN-1 group was administered 4 mg/kg HN-1 every other day for 14 days. The tumor volume was measured daily with a quantitative value of [length × width^2^]/2. Finally, we euthanized the mice, weighed the tumors, and collected primary tumor cells for a flow cytometry analysis.

### CD8^+^ T cell depletion

When the long and short diameters of the tumors on both sides reached 5 mm, 200 µg anti-CD8α antibody started to be intraperitoneally administered every 4 days to deplete CD8^+^ T cells, as previously reported [22].

### Flow cytometry analysis

Briefly, 1 × 10^6^ cells were suspended in 100 µL of PBS and incubated at room temperature for 30 min with CRT-AF488 (92,516, Abcam), HLA-A2-FITC (343,304, Biolegend), CD11c-APC (301,614, Biolegend), HLA-DR-PerCP-Cy5.5 (307,630, Biolegend), CD80-FITC (305,206, Biolegend), CD86-PE (305,406, Biolegend), and CD83-APC (305,312, Biolegend) antibodies.

Mouse primary tumor cells were chopped and digested with the EZ enzyme (Nitta Gelatin Company, Osaka, Japan) to obtain single-cell suspensions. Then, the single-cell suspensions were stained with TruStain FcX (anti-mouse CD16/32 101,320; Biolegend) to block nonspecific staining. Next, the cells were stained on ice for 30 min using the following combinations: CD45-PerCP (103,130, Biolegend)/CD11b-FITC (101,205, Biolegend)/CD11c-APC (117,309, Biolegend); CD3-FITC (100,203, Biolegend)/CD4-PE (100,407, Biolegend)/CD8a-APC (100,711, Biolegend)/CD45-PerCP; CD3-FITC/CD4-BV785 (100,551, Biolegend)/CD25-PE (102,007, Biolegend)/Foxp3-AF647 (126,407, Biolegend); CD45-perCP/CD11b-FITC/Ly-6G-APC (127,613, Biolegend)/Ly-6 C-BV421 (128,031, Biolegend); and CD45-perCP/CD11b-FITC/F4/80-APC (123,115, Biolegend)/Gr-1-PE (108,407, Biolegend). Treg cells were stained with surface markers and, intracellularly, with Foxp3-AF647 after fixation and permeabilization with the True-Nuclear Transcription Factor Buffer Set (Biolegend). The specific gating strategy is shown in Supplementary Fig. 2. Finally, the samples were detected with BD FACSCelesta (BD Biosciences, San Jose, CA, USA).

### Western blotting

The cells were lysed using the RIPA buffer, and the protein lysates were separated via 10% polyacrylamide gel electrophoresis (SDS–PAGE) and subsequently transferred to a PVDF membrane. Next, the membranes were blocked with 10% skim milk and incubated with primary antibodies overnight at 4 °C. Afterwards, the membrane was washed carefully and incubated with a secondary antibody (goat anti-rabbit IgG-HRP (1:10000, ab6721, Abcam) or goat anti-mouse IgG-HRP (1:15000, ab205719, Abcam)) for one hour at room temperature. A Supersignal West Pico Plus (Thermo Fisher Scientific, Inc.) was used for chemiluminescence, and a Bio-Rad GelDoc XR + system was used for detection (Bio-Rad, Berkeley, CA, USA). The ImageJ (version 5.2.1) software was used to analyze the data.

The primary antibodies used were anti-GAPDH (1:1000, 2118, Cell Signaling Technology), anti-STING (1:1000, 13,647, Cell Signaling Technology), anti-phospho-STING (1:1000, 50,907, Cell Signaling Technology), anti-TBK1 (1:1000, 38,066, Cell Signaling Technology), anti-phospho-TBK1 (1:1000, 5483, Cell Signaling Technology), anti-IRF-3 (1:1000, 4302, Cell Signaling Technology), anti-phospho-IRF-3 (1:1000, 79,945, Cell Signaling Technology) and anti-mouse Phospho-STING (Ser365) (1:1000, 72,971, Cell Signaling Technology).

### Transfection

MDA-MB-231 and MDA-MB-453 cells were transfected with 100 pmol of the following STING-specific siRNAs: si-STING-1, 5’-GCCCUUCACUUGGAUGCUUTT-3’; si-STING-2, 5’-GCAUUACAACAACCUGCUATT-3’; and si-STING-3, 5’-GCAUCAAGGAUCGGGUUUATT-3’. The scrambled RNA 5’-ACGUAACACGCCCGGAGUAGT-3’ was transfected as a control. The STING mRNA and protein levels were detected via quantitative real-time PCR and Western blotting, respectively.

### RNA sequencing

A total of 2 × 10^6^ MDA-MB-231 and MDA-MB-453 cells were treated with 20 µM HN-1 for 24 h, after which the total RNA was extracted with TRIzol. These samples were sequenced at the Novogene Technology Laboratory (Novogene Technology, Inc., Beijing, China). We analyzed the differential gene expression between the two groups based on the DESeq2 R package (1.16.1). The data were subsequently transformed into Venn diagrams and heatmaps. Cluster Profiler R was used to conduct Gene Ontology (GO) and KEGG enrichment analyses of the DEGs.

### Primary breast cancer cell sensitivity test

Drug sensitivity was assessed via a collagen gel droplet-embedded culture drug sensitivity assay (CD-DST, Kurabo, Osaka, Japan), according to the manufacturer’s instructions [23]. Briefly, fresh breast cancer samples were obtained after surgery, minced, and digested. The disassociated cells were cultured in a collagen droplet, which provided a three-dimensional environment for breast cancer cells. Every droplet contained 1-1.5 × 10^3^ cells, with 3 droplets per well, and was treated with 20 µM HN-1 for 24 h in 6-well plate. The culture medium was replaced with prepared culture media-2 (PCM-2, Kurabo) without FBS for an additional 7 days. Viable cells were stained with neutral red and fixed with neutral formalin. After air-drying, cell viability was quantitatively evaluated utilizing ImageJ.

### Statistical analysis

The SPSS software version 21 (IBM Corp., Armonk, NY, USA) was used to conduct the data analysis. Unless otherwise specified, three biological replicates were routinely used in the experiment. The standard deviation of the average values of three independent experiments was represented by the mean ± standard deviation. The two groups were compared with t tests or one-way ANOVA. A *P* value < 0.05 indicated a statistically significant difference (*P < 0.05, **P < 0.01).

## Results

### HN-1 triggered ICD in TNBC cells

**Fig. 1:**
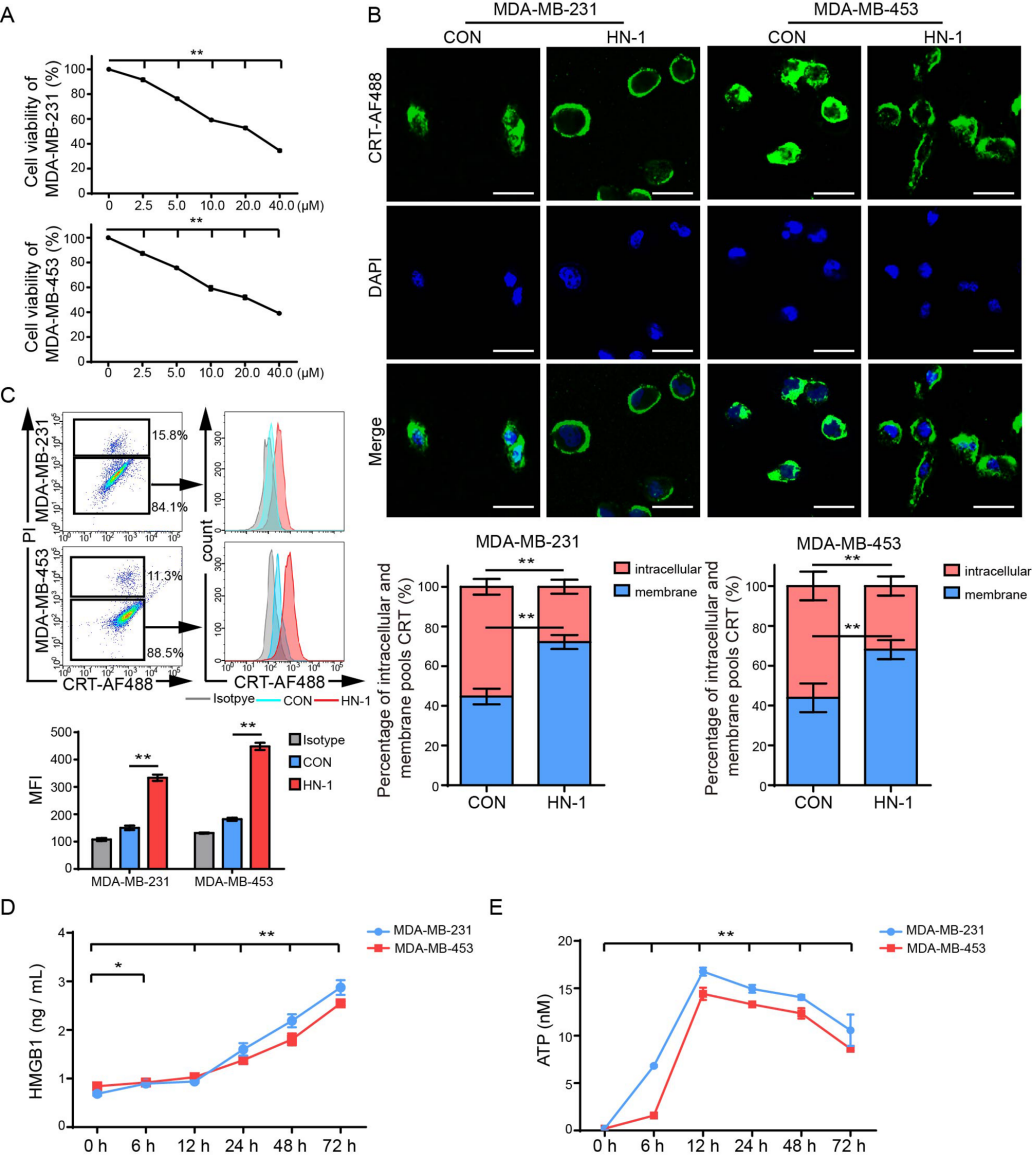
HN-1 treatment triggered ICD in TNBC cells. (**A**) MDA-MB-231 and MDA-MB-453 cells were treated with 0 µM, 2.5 µM, 5 µM, 10 µM, 20 µM, or 40 µM HN-1 for 24 h. Cell viability was determined via a CCK8 assay. (**B**) After 10 h of HN-1 treatment, the cells were immunofluorescently stained with CRT and DAPI and analyzed via confocal microscopy. Scale bar: 20 μm. The percentage of intracellular and membrane CRT staining was analyzed using the ImageJ software. (**C**) CRT and PI were used to stain the cells, which were then gated on a PI-negative population. The cell surface expression of HN-1 treatment-altered CRT was quantified. (**D, E**) The secretion of HMGB1 (**D**) and ATP (**E**) was detected via ELISA and a chemiluminescence assay. The data were acquired from three independent experiments. * *p* < 0.05, ** *p* < 0.01. CON, control

To determine the IC50 of HN-1 against TNBC cell lines, the MDA-MB-231 and MDA-MB-453 cells were treated with different concentrations of HN-1 for 24 h, and the IC50 was determined by a CCK8 assay. As shown in Fig. 1A, HN-1 suppressed TNBC cell growth in a concentration-dependent manner. The IC50 against MDA-MB-231 and MDA-MB-453 cells was 19.1 µM and 21.1 µM, respectively. Therefore, unless otherwise indicated, 20 µM was used in the following experiments. To further confirm the anti-tumor effects of HN-1 on a TNBC in vivo model, MDA-MB-231 cells, or MDA-MB-453 cells were inoculated into BALB/c nude mice mammary fads and treated with 4 mg/kg HN-1 every other day. HN-1 inhibited tumor growth in the nude mice (Supplementary Fig. 3). To investigate whether HN-1 triggered TNBC cell ICD, MDA-MB-231 and MDA-MB-453 cells were treated with HN-1 for 10 h. A DMSO treatment was used in the control group. As shown in Fig. 1B, after HN-1 treatment, the CRT originally expressed in the cytoplasm was released onto the surface of the cell membrane. A quantitative analysis of the fluorescence intensity using ImageJ showed that, compared with the control group, it was significantly greater on the HN-1-treated group’s cell membrane. Cell membrane CRT, a marker of ICD, is an “eat me” signal that stimulates immune activation [24]. We used flow cytometry to further confirm the expression of CRT on the cell surface and removed PI-stained cells to exclude the influence of changes in dead cells’ membrane permeability (Fig. 1C). Compared to the DMSO treatment, the HN-1 treatment significantly increased the fluorescence intensity of CRT on the cell surface. Furthermore, other ICD markers, including HMGB1 and ATP, were monitored after HN-1 treatment. As shown in Fig. 1D, the HN-1 treatment significantly induced HMGB1 and ATP release in both MDA-MB-231 and MDA-MB-453 cells, as well as DAMP release on murine TNBC 4T1 cells (Supplementary Fig. 4). These data collectively indicated that HN-1 induced ICD in TNBC cells.

### HN-1 treatment induced DC activation

**Fig. 2:**
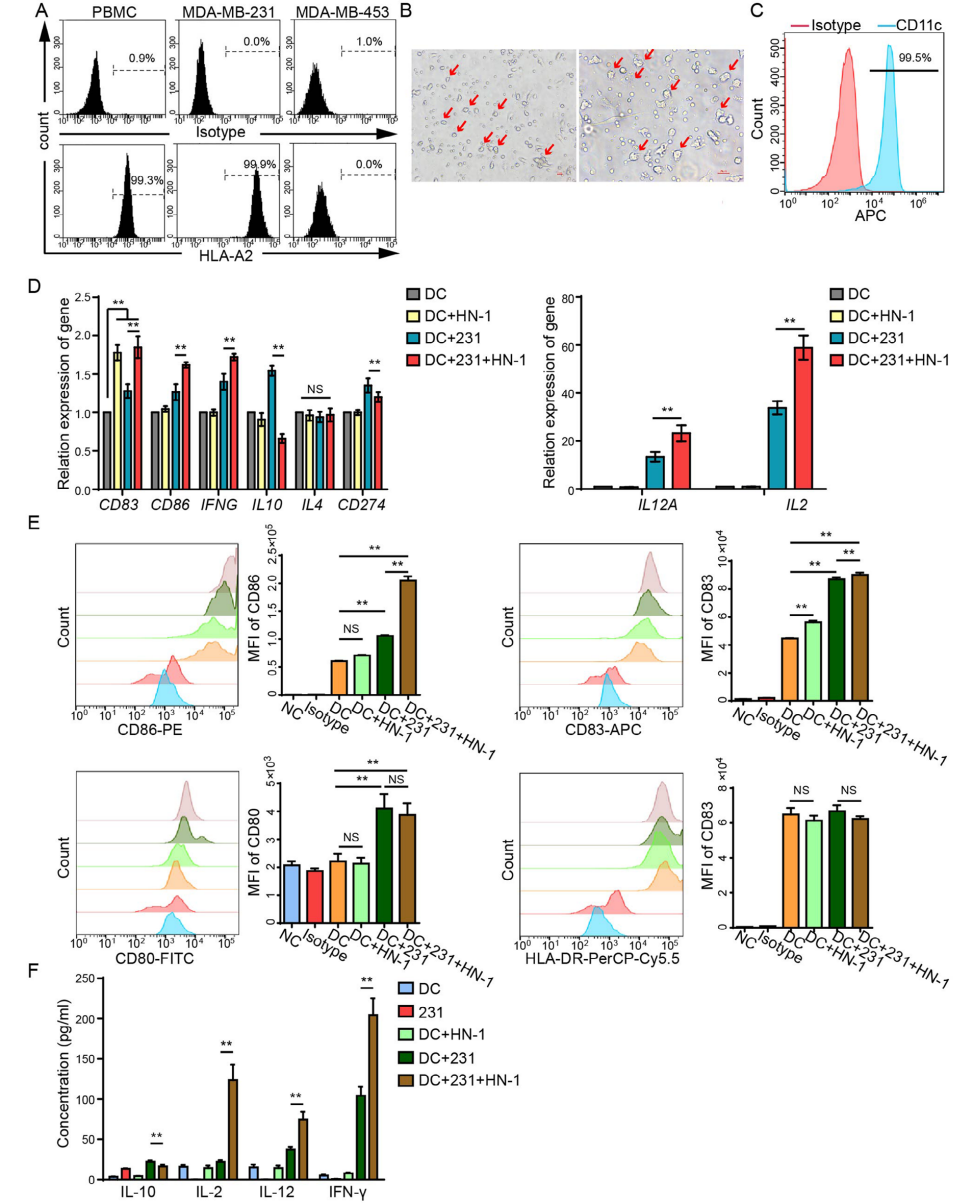
HN-1 treatment induced DC activation. (**A**) PBMCs from healthy volunteers were stained for HLA-A2 and detected via flow cytometry. (**B** and **C**) Collected PBMCs were stimulated with IL-4 and GM-CSF for 5 days. DCs were confirmed based on (**B**) morphological features and (**C**) CD11c expression. (**D**) MDA-MB-231 cells were pretreated with HN-1 for 24 h, co-cultured with DCs for an additional 48 h, and then separated by staining with CD45. CD45^+^ cells were collected as DCs, and the relative expression levels of *CD83*, *CD86*, *IFNG*, *IL10*, *IL4*, *CD274*, *IL12A*, and *IL2* were detected via qRT‒PCR. (**E**) The expression levels of CD80, CD83, HLA-DR, and CD86 were detected via flow cytometry. An isotype antibody was used as a control. The data are presented as the median fluorescence intensity (MFI). (**F**) The culture supernatant was collected, and the concentrations of IL10, IL-2, IL-12, and IFN-γ were detected via ELISA. The data were acquired from three independent experiments. ** *p* < 0.01. NC, negative control

ICD can activate DC function by releasing DAMPs, thereby promoting anti-tumor immune responses [25]. To exclude nonspecific activation due to the different HLAs, HLA-A2-positive MDA-MB-231 cells, consistent with the HLA subtypes of the volunteers’ PBMCs, were selected for subsequent co-culture experiments (Fig. 2A). For DC induction, the PBMCs were stimulated with IL-4 and GM-CSF for 5 days. DCs were confirmed based on both morphological features and CD11c expression. A total of 99.37 ± 0.15% of the cells were confirmed to be DCs (Fig. 2B and C). To investigate whether HN-1-triggered ICD could activate DC function, the DCs were co-cultured with HN-1-pretreated MDA-MB-231 cells for 48 h and then separated based on CD45 expression using a cell sorter. The DC activation and maturation markers were determined using qRT‒PCR. As shown in Fig. 2D, the HN-1 treatment did not affect the expression levels of the *CD86*, *IFNG*, *IL10*, *IL4*, *IL12A*, or *IL2* genes in DCs and slightly enhanced *CD83* expression in DCs. When the DCs were co-cultured with pretreated MDA-MB-231 cells, *CD86*, *CD83*, *IL12A*, *INFG*, and *IL2* expression was enhanced, while *CD274* and *IL10* expression was downregulated. The expression levels of CD80, CD86, CD83, and HLA-DR were further confirmed via flow cytometry. Compared with a co-culture with MDA-MB-231 cells without HN-1 stimulation, the DC co-culture with HN-1-pretreated MDA-MB-231 cells enhanced CD86 and CD83 expression (Fig. 2E). The cytokine secretion of the cell culture supernatants was compared (Fig. 2F). In line with the findings shown in Fig. 2C, the co-cultivation of DCs with MDA-MB-231 cells pretreated with HN-1 resulted in an increase in IL-2, IL-12, and IFN-γ secretion while reducing the secretion of IL-10. These data suggest that HN-1-induced ICD activates DC functions.

### HN-1 increased immune cell infiltration in a mouse tumor model

**Fig. 3:**
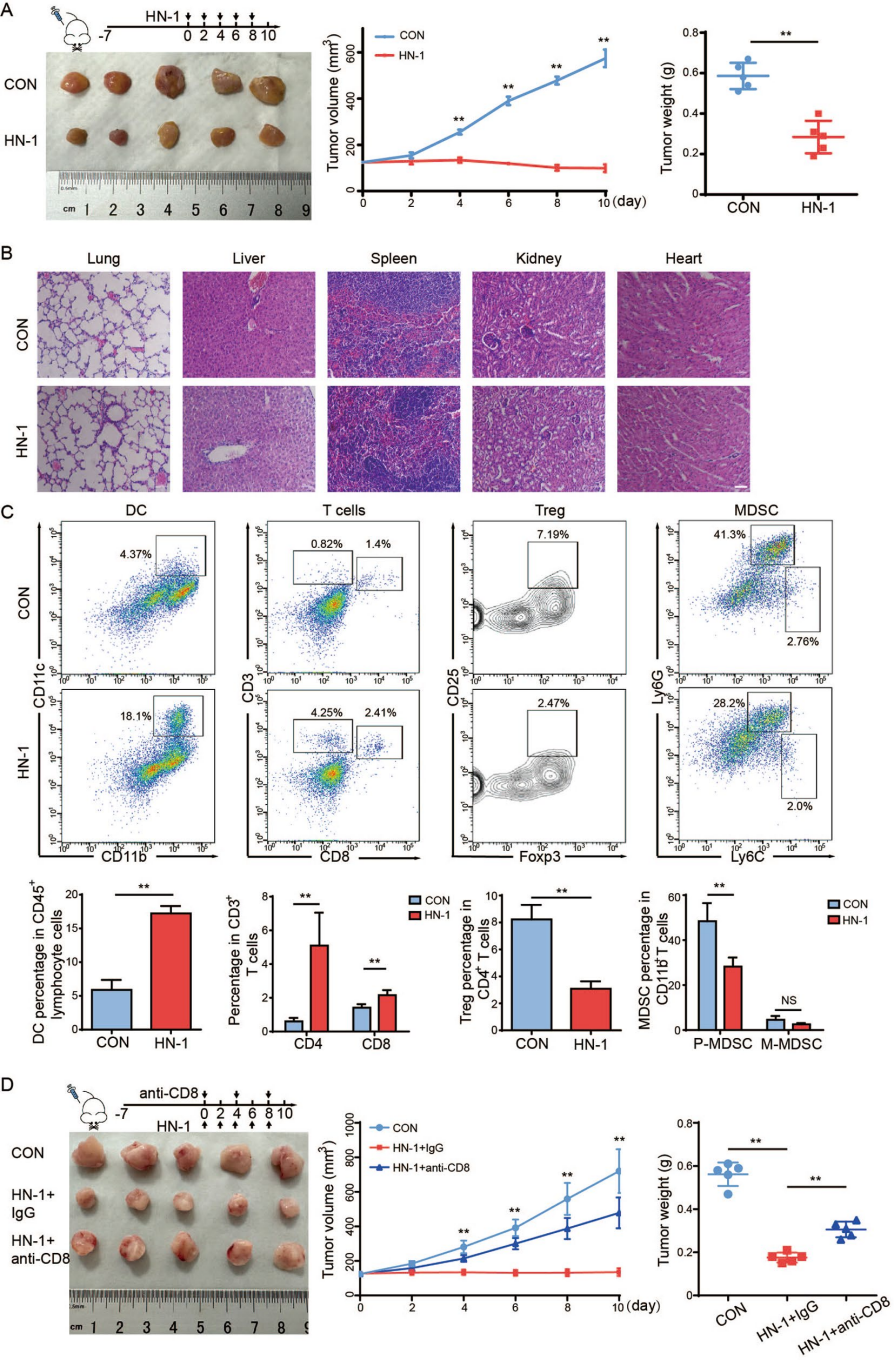
HN-1 increased immune cell infiltration in the immunocompetent mice tumor model. BALB/c mice were inoculated into mammary fat pads with 4T1 cells. PBS or 4 mg/kg HN-1 was intraperitoneally administered every other day for 14 days. *n* = 5/group. (**A**) Tumor volume was quantified daily and is expressed as the mean ± SD. (**B**) The hearts, livers, spleens, lungs, and kidneys after the HN-1 treatment were stained with hematoxylin and eosin. Scale bar = 50 μm. (**C**) Primary tumor cells were collected from tumor-bearing mice and stained with DC, T cell, Treg cell, and MDSC panels. DC panel: CD45, CD11b, and CD11c; T cell panel: CD45, CD3, and CD8; Treg cell panel: CD45, CD3, CD4, CD25, and Foxp3; and MDSC panel: CD45, CD11b, Ly6G, and Ly6C. The proportions of these tumor-infiltrating immune cells are expressed as the mean ± SD. (**D**) Mice experimental design for CD8^+^ T cell depletion. The mice were divided into three groups: PBS + IgG, HN-1 + IgG, and HN-1 + anti-CD8α. A total of 200 µg anti-CD8α antibody started to be intraperitoneally administered every 4 days to deplete the CD8^+^ T cells. A total of 4 mg/kg HN-1 was intraperitoneally administered every other day for 14 days. *n* = 5/group. The tumor volume was quantified daily and is expressed as the mean ± SD. ** *p* < 0.01. CON, control

BALB/c-derived 4T1 cells, along with EMT6 cells, are usually used for TNBC studies in immunocompetent murine orthotopic models [26]. To study the effects of HN-1 on immune activation in vivo, 4T1 cells were implanted into BALB/c mouse mammary fat pads. Seven days after inoculation, the tumor-bearing mice were divided into two groups and intraperitoneally injected with 4 mg/kg HN-1 or PBS as a control for the following fourteen days. Consistent with our previous observations, HN-1 significantly suppressed tumor growth in vivo (Fig. 3A) [20]. The side effects of HN-1 were further evaluated by staining the main organs (hearts, livers, spleens, lungs, and kidneys) with hematoxylin and eosin, and the results showed that the HN-1 treatment had no pathological toxicity or adverse reactions (Fig. 3B). Primary tumor cells were collected to detect alterations in the immune microenvironment after HN-1 treatment. As shown in Fig. 3C, after HN-1 treatment, the proportion of anti-tumor immune cells (DCs and CD4^+^ and CD8^+^ T cells) increased, but the percentage of immunosuppressive Treg cells decreased. The effects on CD4^+^ T cells were greater than those on CD8^+^ T cells. MDSCs are abundant in primary tumor tissues and inhibit T cell activity [27]. They are divided into two main subpopulations with distinct morphological and functional properties: monocyte MDSCs (M-MDSCs) and PMN-MDSCs. In mice, M-MDSCs and PMN-MDSCs are defined as CD11b^+^ Ly6G^−^ Ly6C^hi^ and CD11b^+^ Ly6G^+^ Ly6C^lo^, respectively. In our study, HN-1 treatment decreased the frequency of PMN-MDSCs in the TME but not that of M-MDSCs. In another in vivo study with EMT6-bearing BALB/c mice, similar HN-1 effects on immune activation were observed, where, in addition to increasing DC and T cell infiltration within the TME, PMN-MDSCs were decreased (Supplementary Fig. 5). These data indicate that HN-1-induced ICD alters both the lymphoid and myeloid TME in tumor-bearing mice.

ICD activates DC function and promotes tumor antigens’ presentation to CD8^+^ T cells. In order to prove that CD8^+^ T cells are necessary for HN-1-mediated immunogenic cell death, we examined the effects of CD8^+^ T cell depletion on HN-1-mediated anti-tumor response (Fig. 3D). After the long and short diameters of the tumors reached 5 mm, the mice were divided into three groups—HN-1, HN-1 + anti-CD8α, or control IgG—for another 14 days. Compared to the tumor growth curve in the HN-1 treatment group, HN-1 + anti-CD8 significantly increased the tumor growth rate. Similar results were also observed in the tumor weight comparisons (Fig. 3D). These results demonstrated that CD8^+^ T cell depletion abrogated HN-1-mediated tumor growth inhibition, indicating that the immune cells were necessary for the anti-tumor effect of HN-1.

### HN-1 induced ICD via the STING signaling pathway

**Fig. 4:**
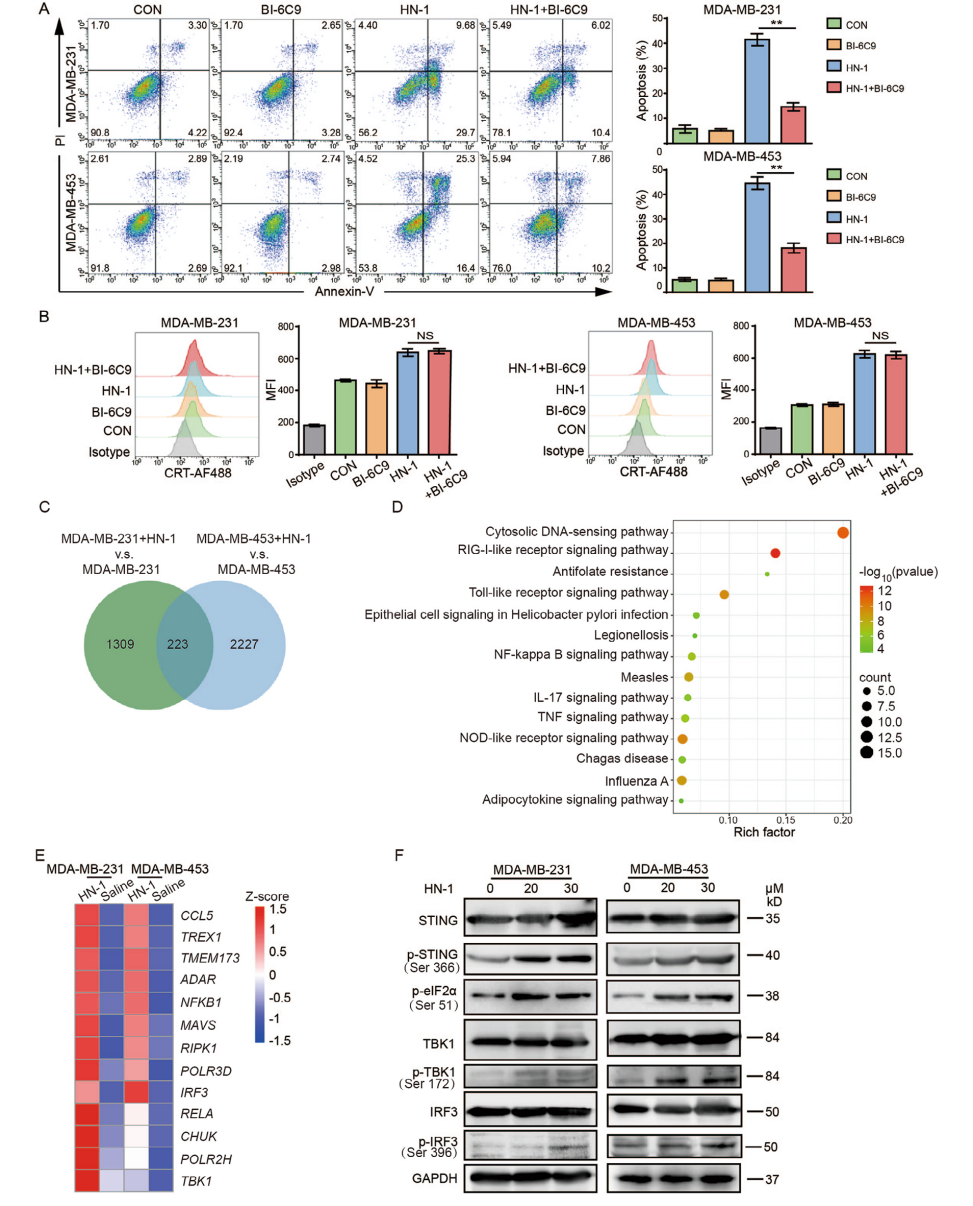
HN-1 treatment induced ICD via the cGAS-STING signaling pathway. MDA-MB-231 and MDA-MB-453 cells were pretreated with 10 µM BI-6C9 for 10 h, then treated with 20 µM HN-1 for a further 24 h. (**A**) Cell apoptosis was detected by staining with Annexin-V and PI. (**B**) CRT exposure on PI-negative cells was detected by flow cytometry. The data were acquired from three independent experiments. *n* = 3/group. (**C**) The common DEGs related to the HN-1 treatment in both MDA-MB-231 and MDA-MB-453 cells are shown in the Venn diagram. (**D**) The common DEGs were enriched according to a KEGG pathway analysis. (**E**) Z scores of the mean TPM values for each sample are shown in the heatmap. (**F**) The phosphorylation of cGAS-STING pathway proteins was detected via Western blotting

According to our previous studies, HN-1 induces apoptosis through the AIF (apoptosis-inducing factor) pathway. To explore whether HN-1-induced ICD is dependent on AIF, we used BI-6C9, an inhibitor of Bid, suppresses AIF translocation and cell apoptosis [28]. Pretreated the TNBC cells for 10 h with BI-6C9 before treatment with HN-1 significantly inhibited cell apoptosis (Fig. 4A). However, CRT exposure, one of the indicators of ICD, was not affected (Fig. 4B).

We further investigated the mechanism of HN-1-induced ICD in TNBC using HN-1-treated MDA-MB-231 and MDA-MB-453 cells and analyzed the differential mRNA expression before and after treatment. A Venn diagram was constructed, revealing a total of 223 common differentially expressed genes (DEGs) (Fig. 4C). According to the KEGG pathway enrichment analysis, the common DEGs were involved mainly in DNA sensing in the cytosol (Fig. 4D). The gene expression patterns associated with the genes in these pathways before and after treatment are further shown in the heatmap (Fig. 4E). The cGAS-STING pathway’s response to cytosolic DNA plays an important role in pathogen infections, autoimmune diseases, and anti-tumor immune responses [29]. Therefore, HN-1-treated MDA-MB-231 and MDA-MB-453 cells were subjected to Western blotting, and the expression of proteins related to the cGAS-STING pathway was further confirmed (Fig. 4F). Compared the control group, the phosphorylation (p) of STING in the HN-1 treatment group was greater. STING-related downstream indicators, such as IRF3 and TBK1 phosphorylation, were also significantly upregulated. Furthermore, elF2α phosphorylation, a marker of both ER stress and ICD, was increased by the HN-1 treatment. These results indicate that HN-1-induced ICD activates the STING pathway in TNBC cells.

### HN-1 induced DNA damage and dsDNA fragment release within the cytosol

**Fig. 5:**
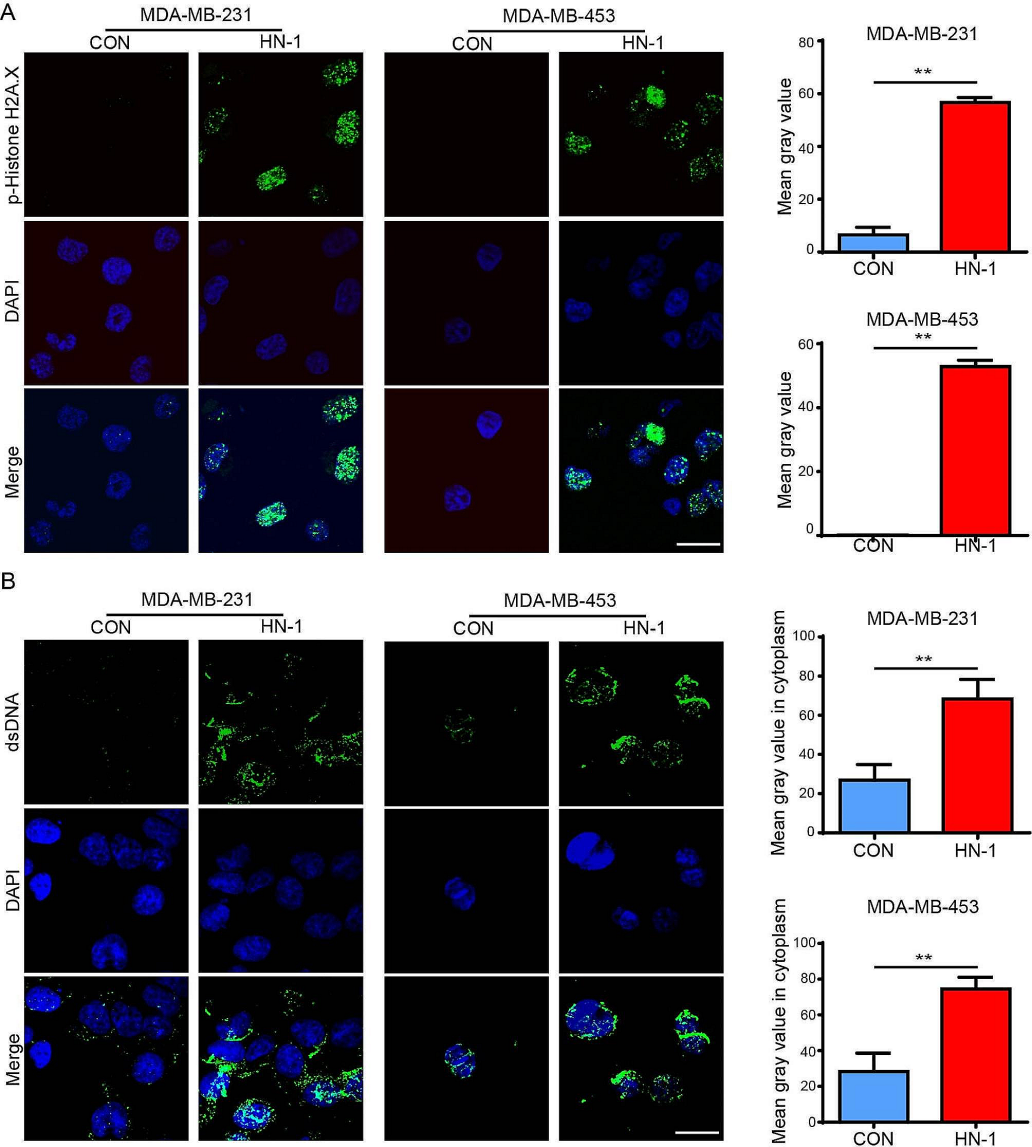
HN-1 induced nuclear damage and dsDNA fragment release in breast cancer cells. (**A** and **B**) MDA-MB-231 and MDA-MB-453 cells were treated with HN-1 for 24 h. The phosphorylation of histone H2A.X (**A**) and dsDNA (**B**) was detected via immunofluorescence staining. The data were acquired from three independent experiments. *n* = 3/group. ** *p* < 0.01. Scale bar: 20 μm. CON, control

Cytoplasm cyclic GMP AMP synthesis (cGAS) is a sensor for dsDNA. Exogenous or endogenous dsDNA binds to cGAS and activates the cGAS-STING pathway to induce ICD [30, 31]. Therefore, we next determined whether HN-1 treatment promoted dsDNA release. In contrast with the control group, HN-1 treatment significantly increased histone H2AX phosphorylation, a key marker of DNA damage (Fig. 5A). After the induction of HN-1, cytosolic dsDNA intensity significantly increased (Fig. 5B). However, the release of mitochondrial DNA within the cytosol could not be confirmed via qRT‒PCR (data not shown). These results indicated that the release of dsDNA within the cytosol caused by the HN-1 treatment triggered the activation of the STING pathway.

### Downregulation of STING inhibited ICD in TNBC

**Fig. 6:**
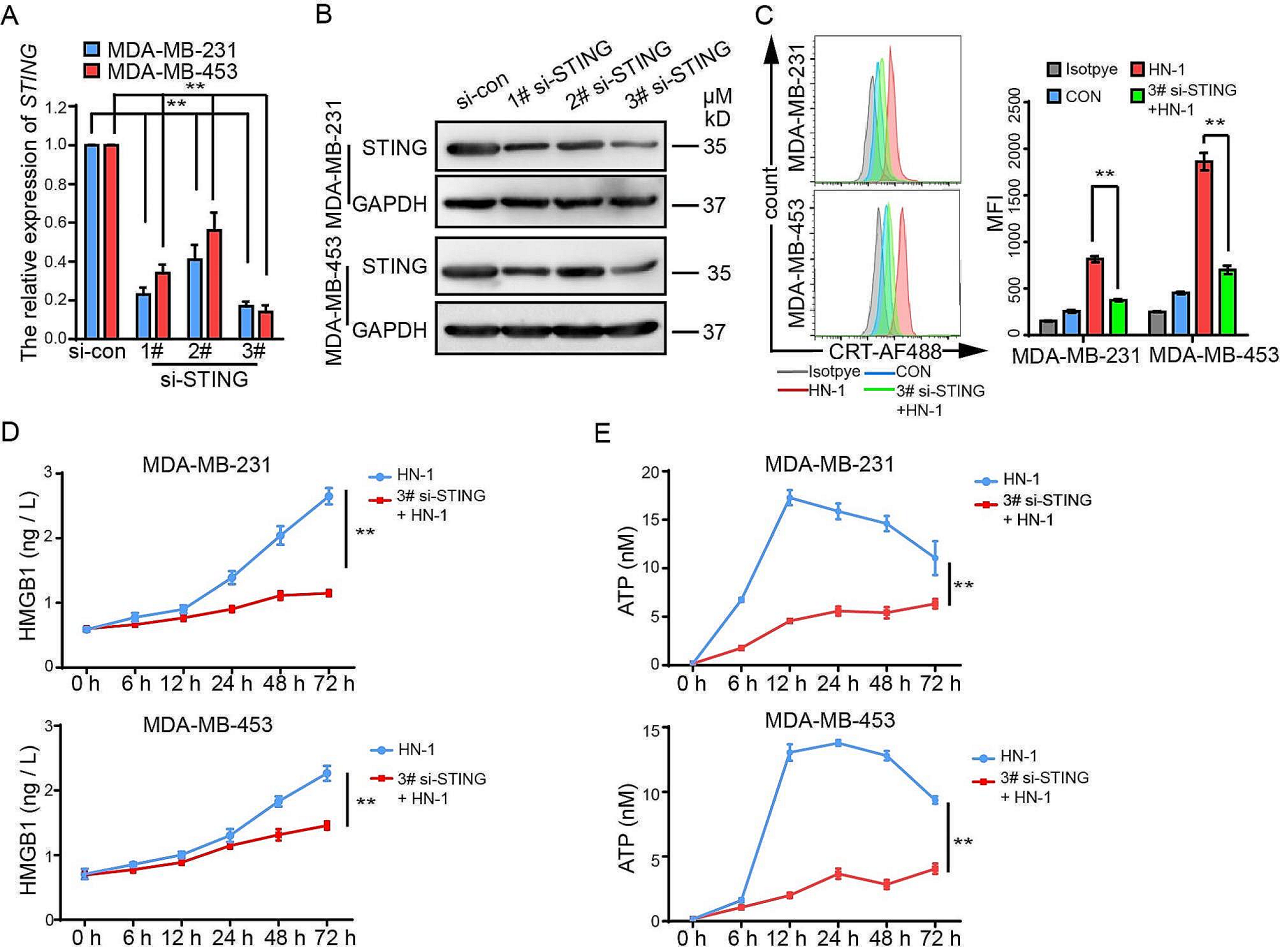
Downregulation of STING inhibited ICD in TNBC. (**A, B**) MDA-MB-231 and MDA-MB-453 cells were transfected with si-con or si-STING. After 24 h of transfection, the mRNA and protein levels of STING were detected via qRT‒PCR (**A**) and Western blotting (**B**), respectively. The transfected cells were treated with HN-1 for an additional 24 h. The cells were stained with PI and CRT. The cell surface expression of CRT was detected via flow cytometry after gating on a PI- population. (**C**) HMGB1 secretion was detected via ELISA. (**D, E**) The secretion of HMGB1 (**D**) and ATP (**E**) was detected via ELISA and a chemiluminescence assay. The data were acquired from three independent experiments and presented as the mean ± SD. *n* = 3/group. ** *p* < 0.01. CON, control

STING is an endoplasmic reticulum (ER) membrane protein that can be activated by cGAS upon dsDNA binding and plays an essential role in anticancer immunity [32]. Next, we investigated whether the knockdown of STING could inhibit HN-1-induced ICD. The si-STING gene was transfected into MDA-MB-231 and MDA-MB-453 cells. The scrambled RNA was transfected as a control, indicated as si-con in Fig. 6. STING was downregulated at both the mRNA and protein levels, especially in 3# si-STING-transfected cells (Fig. 6A and B). We then treated the 3# si-STING-transfected cells with HN-1 for 24 h. Compared to the si-con-transfected cells, CRT cell surface expression was significantly inhibited in the 3# si-STING group (Fig. 6C). Consistent with the CRT results, the release of HMGB1 and ATP, other markers of ICD, was also decreased (Fig. 6D and E). Therefore, these data indicated that the HN-1 treatment triggered ICD through the cGAS-STING pathway.

### HN-1 exhibited inhibitory effects on patient-derived breast cancer cells

**Fig. 7:**
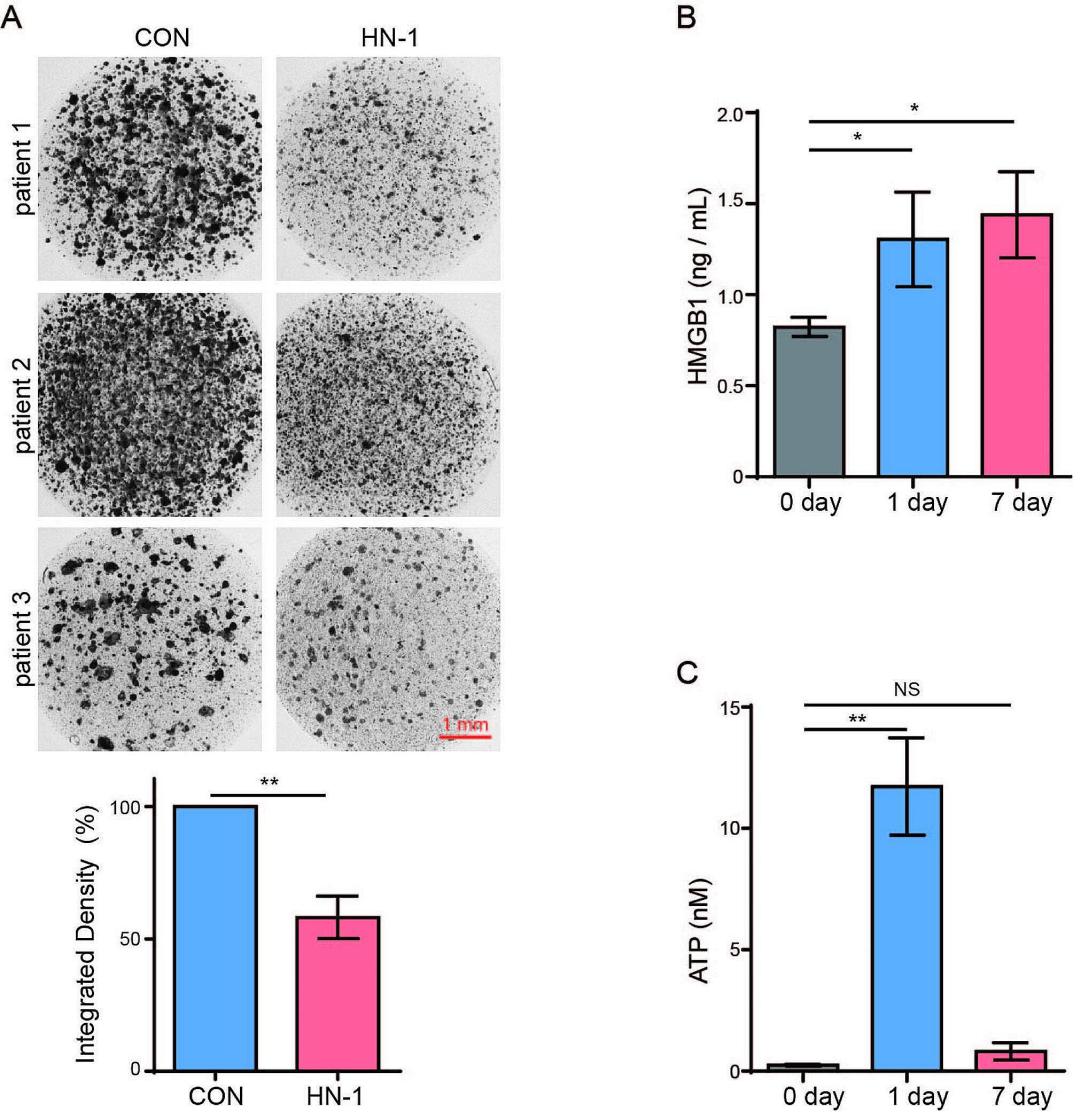
HN-1 exhibited inhibitory effects on patient-derived breast cancer cells. (**A**) Primary breast cancer cells derived from three patients were exposed to 20 µM HN-1. Cells treated with DMSO were used as the controls. *n* = 3 collagen droplets/group. The cells were then stained with neutral red, and cell viability was quantified with ImageJ. Scale bar = 1 mm. (**B, C**) The secretion of HMGB1 (**B**) and ATP (**C**) was detected via ELISA and a chemiluminescence assay. The data are presented as the mean ± SD. * *p* < 0.05, ** *p* < 0.01. Scale bar: 1 mm. CON, control

To investigate the potential clinical effects of HN-1, primary cancer cells obtained from three patients with breast cancer were treated with HN-1 and assessed via a CD-DST assay, an in vitro anticancer drug sensitivity test whose results showed an approximately 91% correlation with clinical efficacy [33, 34]. Cells with a high viability were stained in the dark. Compared to the DMSO-treated cells, the HN-1-treated cells were substantially stained, which indicated the inhibitory effect of HN-1 on primary breast cancer cells (Fig. 7A). To verify that HN-1 induce ICD in primary breast cancer cells, culture medium was collected 1 day and 7 days after HN-1 treatment, and the secretions of ATP and HMGB1 in the supernatant were detected. Compare to 0 day, HMGB1 was slightly increased (Fig. 7B). The secretion of ATP was increased after 24 h of treatment, but was particularly low in the culture supernatant after 7 days (Fig. 7C). These data demonstrated that HN-1 exhibited anti-tumor effects on patient-derived breast cancer cells. However, more data is needed for validation.

## Discussion

HDPs are widely present in various organisms and are important effector molecules for host resistance to external microbial invasion and tumors [35]. Our previous study demonstrated that HN-1, an HDP identified from Hainan frog skin, inhibited the proliferation of breast cancer cells and remodeled the TME through an unknown mechanism [20]. In the current study, we first found that HN-1 treatment induced CRT translocation to the cell surface, which generated an “eat-me” signal for DCs. Indeed, the co-culture of DCs with HN-1-pretreated cancer cells significantly promoted CD83 and CD86 expression. Along with the co-stimulatory signal, the concentrations of secreted IL-2, IL-12, and IFN-γ, immune-active cytokines, increased as well. The expression of ATP, another DAMP which functions as a “find-me” signal to recruit DCs to dying cells, was also enhanced by the HN-1 treatment. These data suggest that HN-1 induces tumor cell ICD and activates DC functions. In line with our in vitro findings, tumor-bearing mice treated with HN-1 exhibited increased DC and CD8^+^ T cell infiltration and the decreased infiltration of immunosuppressive cells such as Treg cells and MDSCs. Therefore, HN-1-triggered ICD remodeled both the lymphoid and myeloid compartments of the TME in tumor-bearing mice. Our previous study has shown that HN-1 caused pore-formation and induced apoptosis in breast cancer through AIF (apoptosis-inducing factor) pathway [20]. AIF, a mitochondrial-localized protein, translocated from the mitochondria to the nucleus to cause DNA cleavage after HN-1 treatment. However, ICD was not affected by pre-treatment with BI-6C9, the inhibitor for AIF translocation. Therefore, based on our previous and the results in this study, dsDNA release might be caused by the pore-formation ability of HN-1. We need to conduct more experiments in the future to confirm this point.

Cytosolic cGAS, an inherent nucleic acid receptor in cells, can sense the release of DNA from pathogens, chromatin, or mitochondria caused by the invasion of microorganisms or cell death into the cytoplasm [36, 37]. 2′,3′-Cyclic GMP AMP (cGAMP) functions as a second messenger of cGAS, binds to the ER-associated protein STING, and helps the transfer of STING to the Golgi apparatus to bind with TBK1 and IκB kinase-ε (IKKε) to form the STING signalosome. The STING signalosome, via the recruitment and activation of the canonical interferon regulatory factor 3 (IRF3) and type I interferon pathways, plays an essential role in the regulation of adaptive immune responses, tissue repair and regeneration, host defense, and autoimmune disorders [38–41]. STING agonists, effective immune adjuvants, are expected to be used in the future to treat tumors [42, 43]. In addition to its functions in immune cells, the activation of the cGAS-STING signaling pathway in tumor cells has been shown to induce anti-tumor immune responses in several studies [44]. Several studies have demonstrated that the activation of the cGAS-STING pathway promotes DC maturation and induces cytotoxic lymphocytes’ infiltration in breast cancer [45–47]. In addition to the canonical STING/TBK1/IRF3 pathway, nonclassical STING pathways play a role in autophagy induction, cell aging, and cell death [48, 49]. One of the nonclassical STING/PERK/eIF2α pathways has been reported to regulate cellular aging and tissue fibrosis [50]. Our previous study demonstrated that the ER stress-associated PERK/eIF2α pathway plays an important role in ICD induction [51].

Due to the lack of ER, PR, and HER2/neu HER2 expression, patients with TNBC cannot benefit from endocrine or anti-HER2-targeted therapy. Distinct from other breast cancer subtypes, TNBC shows higher PD-L1 expression and immune cell infiltration, indicating the immunotherapy potential for TNBC. However, the KEYNOTE-119 phase III study showed no significant improvement in patients with metastatic TNBC in the pembrolizumab monotherapy group compared to the chemotherapy group [52]. Studies have reported that TILs, especially CD8^+^ T cell infiltrates, are relevant to the ICI response rates in patients with TNBC [53, 54]. Combining ICD-triggering therapy with ICIs may be more effective for treating tumors with lower TIL infiltration. ICD-triggering radiation therapy or chemotherapy combined with ICIs has demonstrated increased anti-tumor efficacy [55, 56]. Therefore, further work needs to be conducted to determine whether HN-1 combined therapies could augment immunotherapy efficacy.

## Conclusions

Our study demonstrated that HN-1 could be utilized as a potential compound to augment immunotherapy effects in patients with TNBC.

### Electronic supplementary material

Below is the link to the electronic supplementary material.

**Supplementary Material 1**: Fig. 1. The purity and identity of HN-1 was confirmed by (A) reverse-phase high-performance liquid chromatography and (B) electronic spray ionization mass spectrometry

**Supplementary Material 2**: Fig. 2. Flow cytometry gating strategy for analyzing TME in tumor bearing mice. (A) DCs are represented as CD45^+^, CD11b^+^, and CD11c^+^. (B) T cells are represented as CD45^+^ and CD3^+^ and further divided into CD8^+^ T cells and CD4^+^ T cells according to surface expression. (C) Treg cells are represented as CD3^+^, CD4^+^, CD25^+^, and Foxp3^+^. (D) PMN-MDSCs are represented as CD11b^+^, Ly6G^+^ and Ly6C^−^, and M-MDSCs are CD11b^+^, Ly6G^+^, and Ly6C^+^

**Supplementary Material 3**: Fig. 3. HN-1 inhibited tumor growth in a nude mice tumor model. BALB/c nude mice were inoculated into mammary fat pads with (A) MDA-MB-231 or (B) MDA-MB-453 cells. PBS or 4 mg/kg HN-1 was intraperitoneally administered every other day for 14 days. The tumor volume was quantified daily and is expressed as the mean ± SD. *n* = 4/group. ** *p* < 0.01. CON, control

**Supplementary Material 4**: Fig. 4. HN-1 induced DAMP release in 4T1 cells. (A) 4T1 cells were treated with 10 µM HN-1 for 10 h. The cells were immunofluorescently stained with CRT and DAPI and analyzed via confocal microscopy. Scale bar: 20 μm. The percentage of intracellular and membrane CRT staining was analyzed using the ImageJ software. (B) CRT and PI were used to stain the cells, which were then gated on a PI-negative population. The cell surface expression of CRT altered by the HN-1 treatment was quantified. (C, D) The secretion of HMGB1 (C) and ATP (D) was detected via ELISA and a chemiluminescence assay. The data were acquired from three independent experiments. ** *n* = 3/group. *p* < 0.01. CON, control

**Supplementary Material 5**: Fig. 5. HN-1 increased immune cell infiltration in the EMT6-bearing mice model. BALB/c mice were inoculated into mammary fat pads with EMT6 cells. PBS or 4 mg/kg HN-1 was intraperitoneally administered every other day for 14 days. *n* = 5/group. (A) The tumor volume was quantified daily and is expressed as the mean ± SD. (B) Primary tumor cells were collected from tumor-bearing mice and stained with DC, T cell, Treg cell, and MDSC panels. DC panel: CD45, CD11b, and CD11c; T cell panel: CD45, CD3, and CD8; Treg cell panel: CD45, CD3, CD4, CD25, and Foxp3; and MDSC panel: CD45, CD11b, Ly6G, and Ly6C. The proportion of these tumor-infiltrating immune cells is expressed as the mean ± SD. ** *p* < 0.01. CON, control

## Data Availability

No datasets were generated or analysed during the current study.

## Abbreviations

TNBC: Triple-negative breast cancer
HDP: Host defense peptide
ICD: Immunogenic cell death
DAMPs: Damage-associated molecular patterns
IL: Interleukin
DC: Dendritic cell
ER: Estrogen receptor
PR: Progesterone receptor
HER2: Human epidermal growth factor receptor 2
OS: Overall survival
CTL: Cytotoxic T lymphocyte
CRT: Calreticulin
HSPs: Heat shock proteins
HMGB1: High mobility group box 1
TNF: Tumor necrosis factor
IFN: Interferon
Treg: Regulatory T
dsDNA: Double-strand DNA
PMN-MDSCs: Polymorphonuclear myeloid-derived suppressor cells
PBMCs: Peripheral blood mononuclear cells
HLA: Human leukocyte antigen
ELISAs: Enzyme-linked immunosorbent assays
SDS–PAGE: Sodium dodecyl sulphate polyacrylamide gel electrophoresis
GO: Gene Ontology
CD-DST: Collagen gel droplet-embedded culture drug sensitivity assay
D: Three-dimensional
MDSCs: Monocyte myeloid-derived suppressor cells
DEGs: Differentially expressed genes
P: Phosphorylation
cGAS: Cyclic GMP AMP synthesis
ER: Endoplasmic reticulum
cGAMP: Cyclic GMP AMP
IKKε: IκB kinase-ε
IRF3: Interferon regulatory factor 3
MFI: Median fluorescence intensity
