# Supplementary figures and images for: Hainanenin-1, an oncolytic peptide, triggers immunogenic cell death via STING activation in triple-negative breast cancer
Source: Cell Commun Signal. 2024 Jul 5;22:352. doi: 10.1186/s12964-024-01731-6 (PMC11225514; doi:10.1186/s12964-024-01731-6)

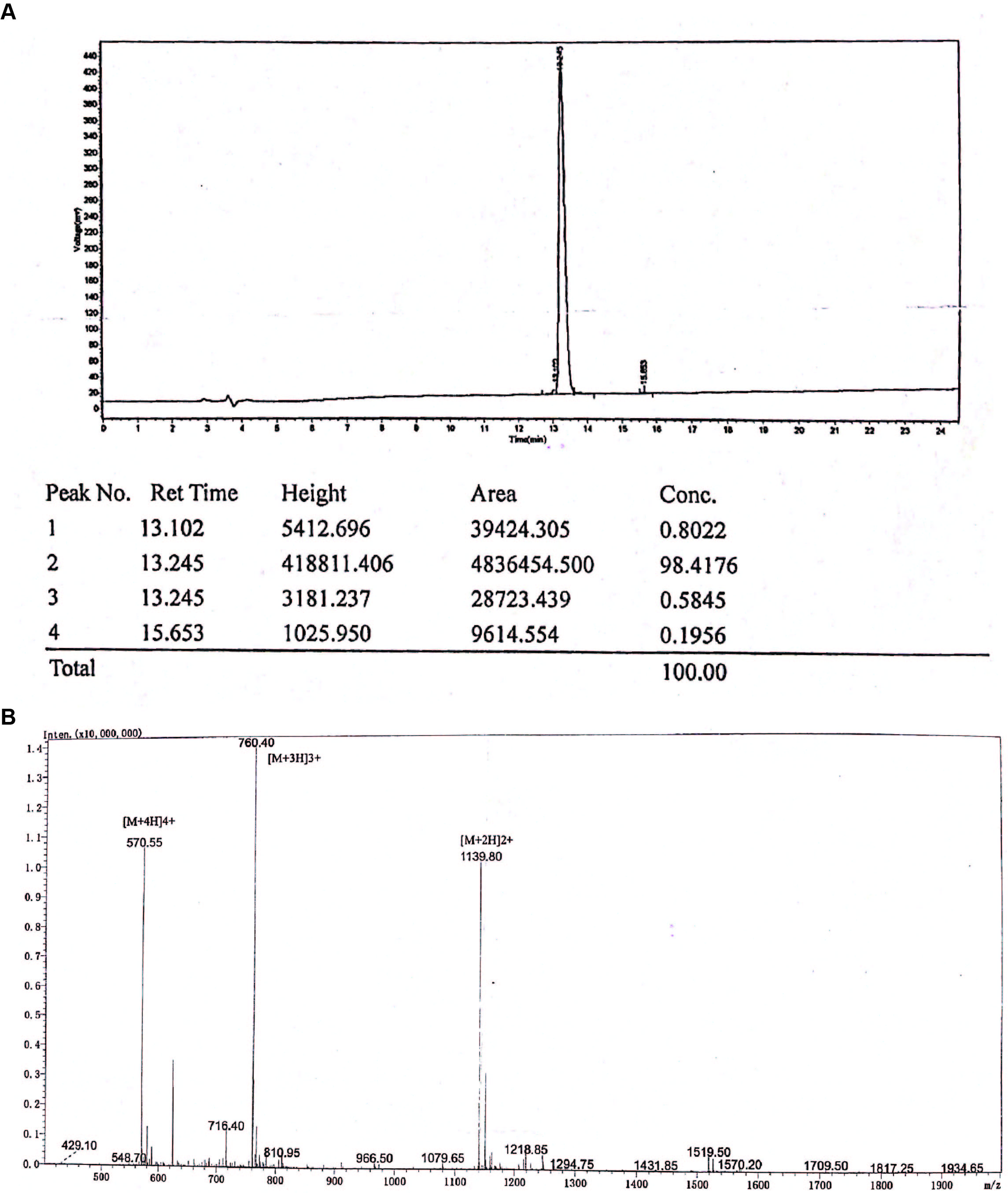

Supplement: Supplementary file 1 — Supplementary Material 1: Fig. 1. The purity and identity of HN-1 was confirmed by (A) reverse-phase high-performance liquid chromatography and (B) electronic spray ionization mass spectrometry [file 12964_2024_1731_MOESM1_ESM.jpg]

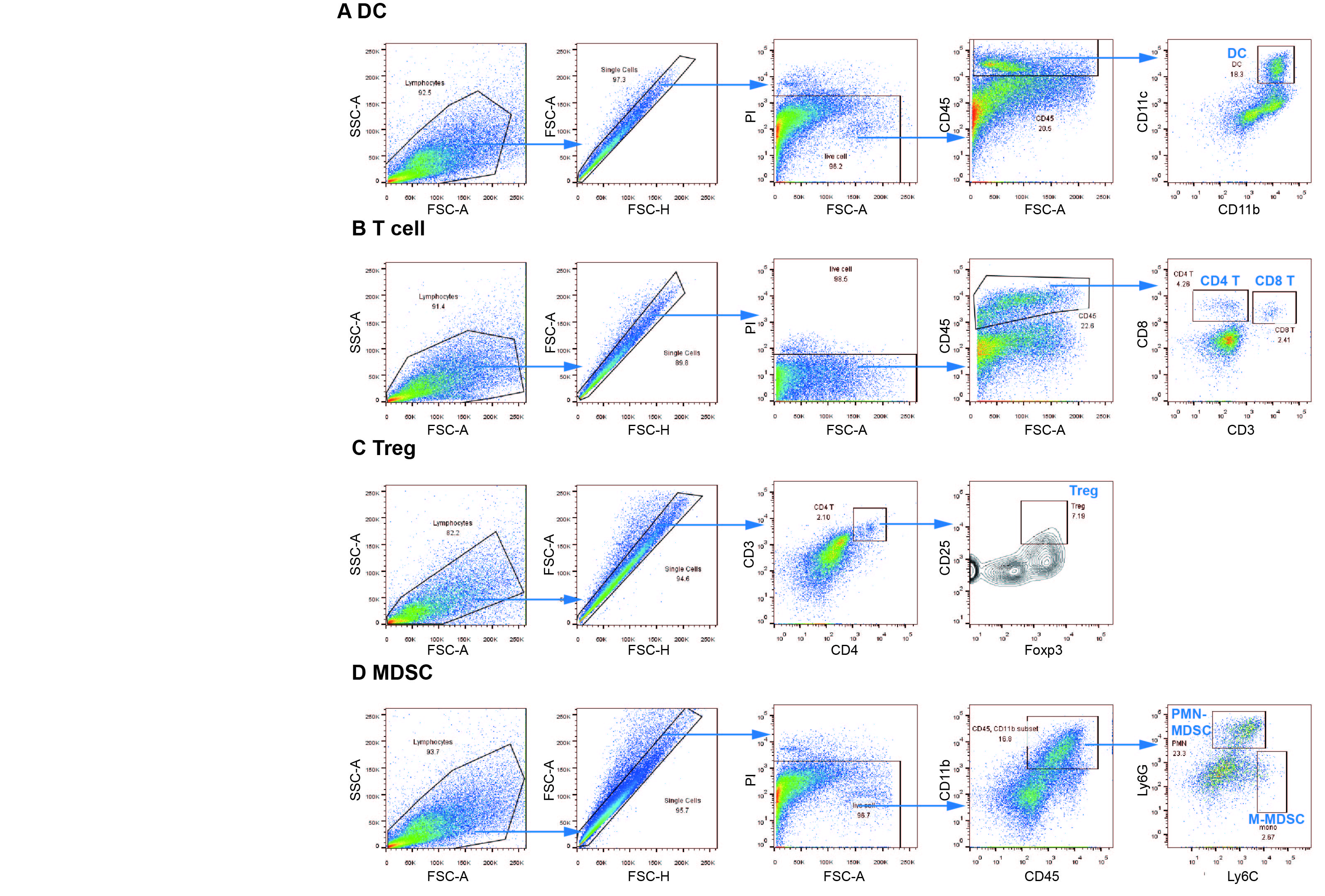

Supplement: Supplementary file 2 — Supplementary Material 2: Fig. 2. Flow cytometry gating strategy for analyzing TME in tumor bearing mice. (A) DCs are represented as CD45+, CD11b+, and CD11c+. (B) T cells are represented as CD45+ and CD3+ and further divided into CD8+ T cells and CD4+ T cells according to surface expression. (C) Treg cells are represented as CD3+, CD4+, CD25+, and Foxp3+. (D) PMN-MDSCs are represented as CD11b+, Ly6G+ and Ly6C−, and M-MDSCs are CD11b+, Ly6G+, and Ly6C+ [file 12964_2024_1731_MOESM2_ESM.jpg]

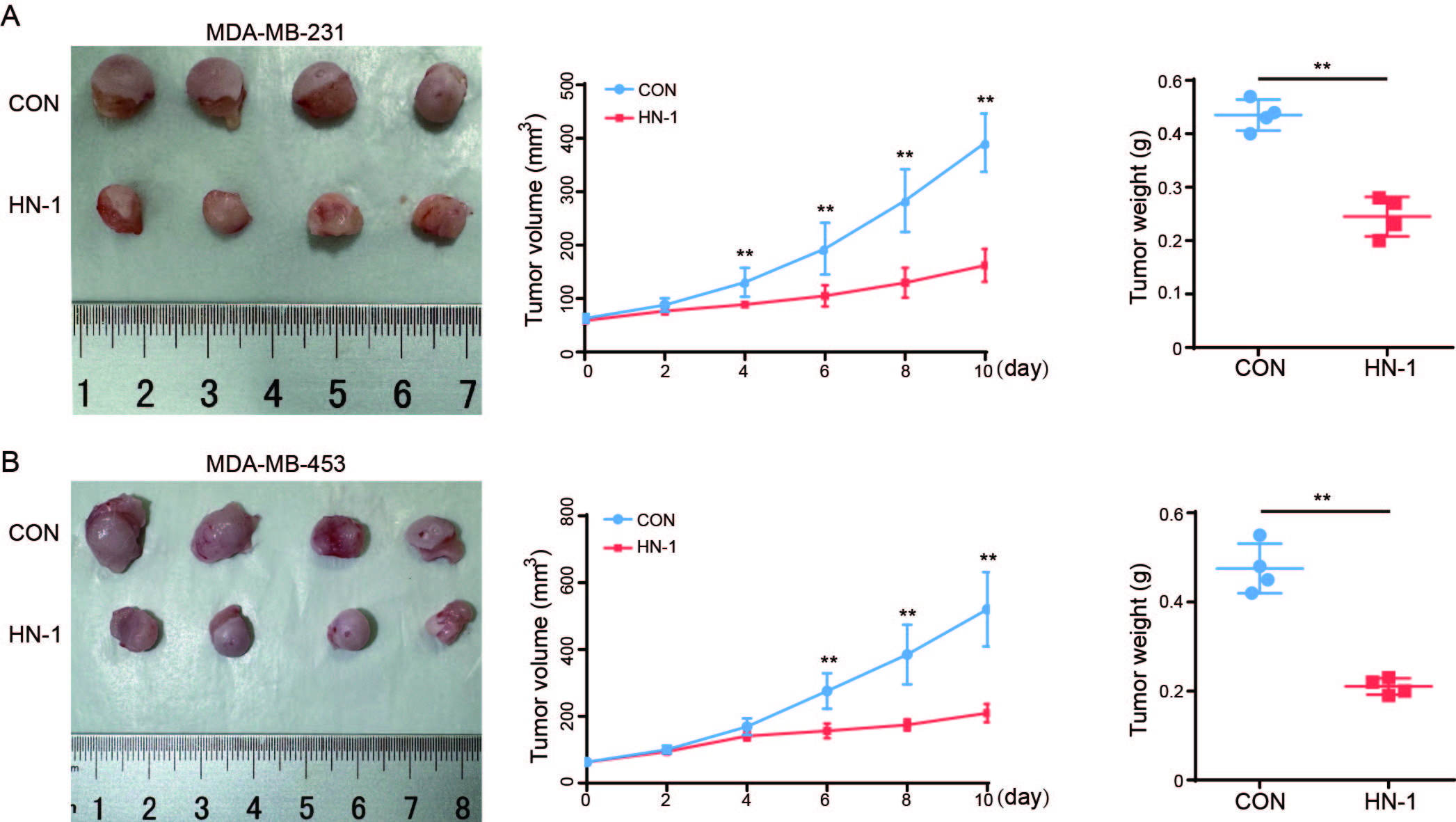

Supplement: Supplementary file 3 — Supplementary Material 3: Fig. 3. HN-1 inhibited tumor growth in a nude mice tumor model. BALB/c nude mice were inoculated into mammary fat pads with (A) MDA-MB-231 or (B) MDA-MB-453 cells. PBS or 4 mg/kg HN-1 was intraperitoneally administered every other day for 14 days. The tumor volume was quantified daily and is expressed as the mean ± SD. n = 4/group. ** p < 0.01. CON, control [file 12964_2024_1731_MOESM3_ESM.jpg]

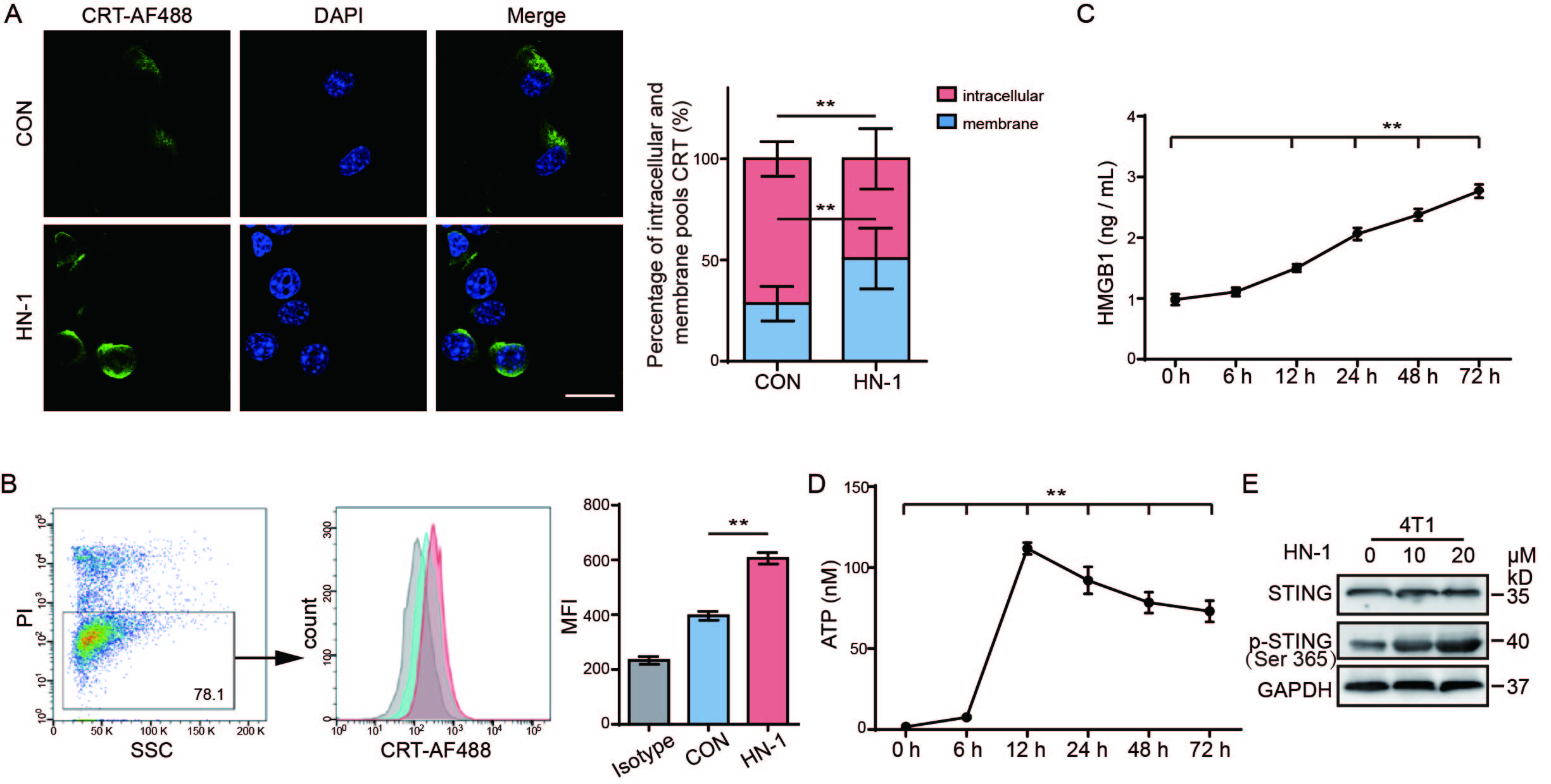

Supplement: Supplementary file 4 — Supplementary Material 4: Fig. 4. HN-1 induced DAMP release in 4T1 cells. (A) 4T1 cells were treated with 10 µM HN-1 for 10 h. The cells were immunofluorescently stained with CRT and DAPI and analyzed via confocal microscopy. Scale bar: 20 μm. The percentage of intracellular and membrane CRT staining was analyzed using the ImageJ software. (B) CRT and PI were used to stain the cells, which were then gated on a PI-negative population. The cell surface expression of CRT altered by the HN-1 treatment was quantified. (C, D) The secretion of HMGB1 (C) and ATP (D) was detected via ELISA and a chemiluminescence assay. The data were acquired from three independent experiments. ** n = 3/group. p < 0.01. CON, control [file 12964_2024_1731_MOESM4_ESM.jpg]

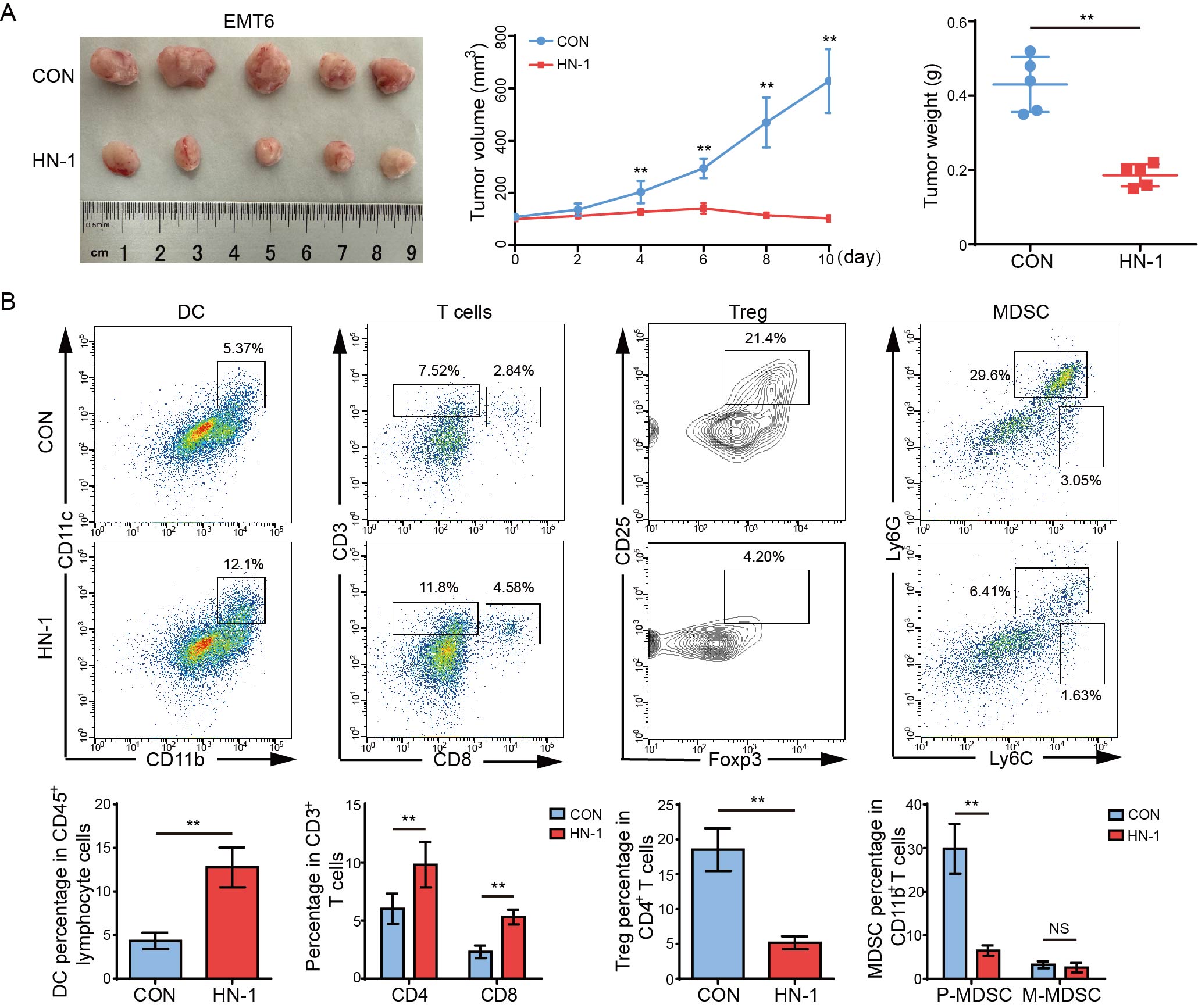

Supplement: Supplementary file 5 — Supplementary Material 5: Fig. 5. HN-1 increased immune cell infiltration in the EMT6-bearing mice model. BALB/c mice were inoculated into mammary fat pads with EMT6 cells. PBS or 4 mg/kg HN-1 was intraperitoneally administered every other day for 14 days. n = 5/group. (A) The tumor volume was quantified daily and is expressed as the mean ± SD. (B) Primary tumor cells were collected from tumor-bearing mice and stained with DC, T cell, Treg cell, and MDSC panels. DC panel: CD45, CD11b, and CD11c; T cell panel: CD45, CD3, and CD8; Treg cell panel: CD45, CD3, CD4, CD25, and Foxp3; and MDSC panel: CD45, CD11b, Ly6G, and Ly6C. The proportion of these tumor-infiltrating immune cells is expressed as the mean ± SD. ** p < 0.01. CON, control [file 12964_2024_1731_MOESM5_ESM.jpg]
